# A finite difference scheme for integrating the Takagi–Taupin equations on an arbitrary orthogonal grid

**DOI:** 10.1107/S2053273322004934

**Published:** 2022-07-08

**Authors:** Mads Carlsen, Hugh Simons

**Affiliations:** a Department of Physics, Technical University of Denmark (DTU), Fysikvej, Building 311, 2800 Kgs. Lyngby, Denmark; Deutsches Electronen-Synchrotron, Germany

**Keywords:** dynamical diffraction, X-ray topography

## Abstract

Calculating dynamical diffraction patterns for X-ray diffraction imaging techniques requires numerical integration of the Takagi–Taupin equations. This is usually performed with a simple, second-order finite difference scheme on a sheared computational grid in which two of the axes are aligned with the wavevectors of the incident and scattered beams. This dictates, especially at low scattering angles, an oblique grid of uneven step sizes. Here a finite difference scheme is presented that carries out this integration in slab-shaped samples on an arbitrary orthogonal grid by implicitly utilizing Fourier interpolation. The scheme achieves the expected second-order convergence and a similar error to the traditional approach for similarly dense grids.

## Abstract 2

A finite difference scheme for integrating the Takagi–Taupin equations inside a slab-shaped crystal is demonstrated and tested.

## 1. Introduction

Simulations based on the propagation of coherent wavefronts are becoming an increasingly common tool for the development of X-ray diffraction imaging techniques, where they are regularly used to evaluate the viability of new methods (Pedersen *et al.*, 2018 ▸; Holstad *et al.*, 2022 ▸) and to investigate the effect of experimental errors (Shabalin *et al.*, 2017 ▸; Carnis *et al.*, 2019 ▸). Furthermore, by accurately predicting coherent interference, such simulation methods are particularly relevant given the recent arrival of highly coherent X-ray sources, such as fourth-generation synchrotrons and free-electron lasers.

When simulating the propagation of coherent wavefronts through large and near-perfect single crystals, multiple scattering effects (*i.e.* dynamical diffraction) become important. One often tries to avoid these dynamical effects [even if occasionally they are the subject of interest (Rodriguez-Fernandez *et al.*, 2021 ▸)] by using highly deformed samples, small grains or relying on the ‘weak beam approximation’, *i.e.* measuring at the tails of the rocking curve (Shabalin *et al.*, 2017 ▸; Holstad *et al.*, 2022 ▸). However, in many cases dynamical effects are unavoidable and must be accounted for in the simulation framework by solving the Takagi–Taupin equations (TTEs): a set of coupled, first-order PDEs (partial differential equations) that, in general, must be integrated numerically (Takagi, 1962 ▸; Taupin, 1967 ▸). When numerically integrating the TTEs (in the two-beam case), it is natural to choose a computational grid with two of its axes aligned with the wavevector of the incident and scattered waves (Taupin, 1967 ▸; Authier *et al.*, 1968 ▸). With this approach, the TTEs have previously been solved via finite difference integration on a structured grid of constant (Authier *et al.*, 1968 ▸) or varying (Epelboin, 1981 ▸) step sizes, or by iterative approaches (Bremer, 1984 ▸; Yan & Li, 2014 ▸). However, the use of sheared grids may complicate matters by requiring the use of a connecting interpolation step when the scattering calculation is combined with other numerical methods, for example to generate input for the incident wavefront, to generate the input for the deformed crystal structure, or when the calculated diffraction patterns are further input into simulations of the downstream optics. Approaches using different coordinate systems have typically involved the use of symmetric scattering geometries in which the sheared coordinate system coincides with a rectangular one (Kolosov & Punegov, 2005 ▸; Osterhoff, 2012 ▸), or a finite element approach that can solve the TTEs on an unstructured grid (Honkanen *et al.*, 2018 ▸). These approaches, however, are either geometrically restrictive or require third-party software.

Ideally, one should be able to straightforwardly integrate the TTEs on an orthogonal grid that requires no intermediate interpolation step. In the kinematical case (*i.e.* not incorporating multiple scattering), Li *et al.* (2020 ▸) described a method for carrying out scattering simulations on an orthogonal grid that implicitly uses Fourier interpolation to avoid making cumulative interpolation errors that would otherwise cause such a calculation to fail. Inspired by this approach, we here describe how implicit Fourier interpolation can also be utilized to integrate the dynamical TTEs on an orthogonal grid. Our approach is based on exponential Rosenbrock-type methods (Hochbruck & Ostermann, 2010 ▸) for the numerical integration, and yields a a result similar to the mixed real-space/reciprocal-space methods called ‘multistep methods’ regularly used to model a wide range of optical scenarios (Li *et al.*, 2017 ▸; Hare & Morrison, 1994 ▸). The method we present is applicable for slab-shaped samples (two parallel surfaces and infinite extent in the orthogonal directions) in Laue geometry, making it ideal for simulating X-ray diffraction images from lightly deformed (*i.e.* strained) materials.

## 2. The Takagi–Taupin equations

The most general framework for treating dynamical diffraction from strained crystals involves the TTE (Takagi, 1962 ▸, 1969 ▸; Taupin, 1967 ▸) which, for the two-beam case, are

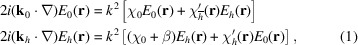

where 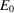 and 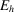 are the complex envelopes of the monochromatic fields of the incident and scattered beams, respectively, 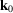 and 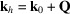 are the vacuum wavevectors of the incident and scattered beams, respectively,^1^ and 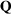 is the scattering vector. 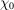 is the average electric susceptibility of the crystal, while 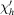 and 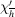 are the spatially varying Fourier components of the electric susceptibility corresponding to the scattering vectors 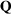 and 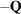 , respectively, given by

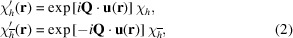

where 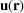 is the displacement field of the crystal. These susceptibility terms are related to the form factors 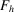 and 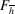 through

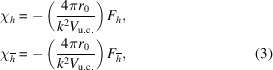

where 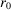 is the classical electron radius and 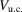 is the volume of the crystal unit cell. Finally, 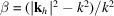 is a measure of the deviation away from the exact Bragg condition, where *k* is the magnitude of 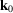 . If we consider only a right-handed ‘rocking’ rotation around an axis parallel to 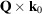 (‘ 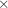 ’ denotes the cross-product) by an angle ϕ, we can write 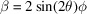 , where 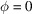 corresponds to the exact satisfaction of the Bragg condition. Rotation of the crystal can also be simulated by the addition of a rotational component to the displacement field (*e.g.* Shabalin *et al.*, 2017 ▸). This alternative approach is also compatible with the method presented here.

If we ignore the scattering terms, the equations (1) are a pair of convection equations whose solution involves the interpolation of the initial condition through the integration volume. Since direct application of a finite difference scheme in a Cartesian coordinate system is equivalent to linear interpolation and would accumulate errors at each step, the traditional approach is to solve the equations in an oblique coordinate system with the axes aligned with the incident and scattered wavevectors. In this way, the interpolation from slice to slice in the computational grid becomes a simple shifting of array elements. Though simple and elegant, the use of such oblique coordinate systems requires additional interpolation steps when the scattering simulation interfaces with material models or other optical simulation steps that require orthogonal grids. The approach we describe here utilizes an orthogonal grid, but avoids cumulative interpolation errors by re-stating the problem in reciprocal space and utilizing Fourier interpolation implicitly.

## 3. Rewriting the TTEs in terms of transverse Fourier transforms

We wish to arrive at a mixed real-/reciprocal-space solution to the TTEs. To achieve this, we first state the TTEs in an orthogonal coordinate system, then Fourier transform the resulting differential equations along the transverse coordinates.

We define an orthogonal grid with the three orthonormal unit vectors 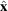 , 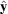 and 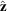 . The only restriction on the choice of coordinate system is that

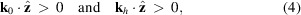

such that the *z* axis takes the role of a quasi-optical axis and we can treat *z* as the dynamical variable and *x* and *y* as transverse variables. To this end, we decompose the vectors 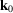 and 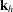 into their *z* components and their projection onto the *x*–*y* plane, *i.e.* 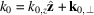 and 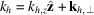 , where 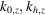 refer to the *z* components of the respective vectors. We can now rewrite equations (1) as

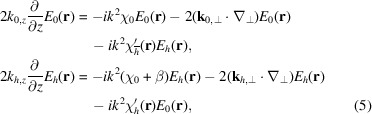

where 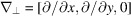 . We now introduce the transverse Fourier transform: 

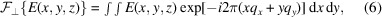

which is inverted by the inverse Fourier transform, appropriately defined as 

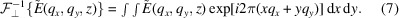

Tildes are used to denote the transforms of functions, namely 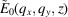 = 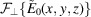 and 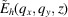 = 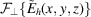 .

With this definition, we Fourier transform the equations (5): 

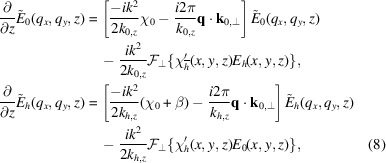

where 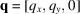 . Here we have assumed that 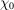 is constant throughout the simulated volume, which limits this approach to slab-shaped crystals. We introduce the angles 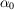 and 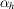 given by 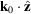 = 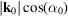 and 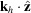 = 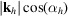 to give

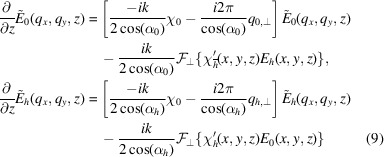

where 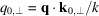 and 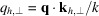 .

In cases where 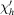 is constant or depends only on *z*, the equations can be solved analytically with Green’s function methods. In the general case where 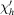 varies as a function of all coordinates, the scattering term 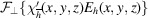 cannot be simplified and finite difference methods must be used.

We note that in cases when both 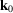 and 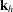 lie within the *x*–*z* plane, the 2D Fourier transforms may be replaced by 1D Fourier transforms along the *x* direction.

## 4. Discretization and numerical evaluation

We introduce a computational grid with axes parallel to those in the coordinate system defined above. It has step sizes 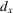 , 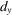 and 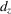 , and number of points 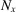 , 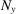 and 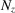 in each dimension. The thickness of the slab-shaped crystal is 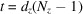 and the simulated volume has side lengths 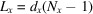 and 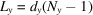 in the transverse directions. A point on the grid 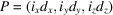 is then indexed by the numbers 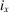 , 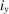 , 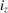 where 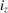 = 0, 1, 2… 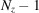 .

In order to utilize discrete Fourier transform methods when solving these equations on a finite grid, we impose zero Dirichlet boundary conditions in the two transverse dimensions, *x* and *y*: 

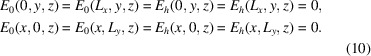

**Figure 1:**
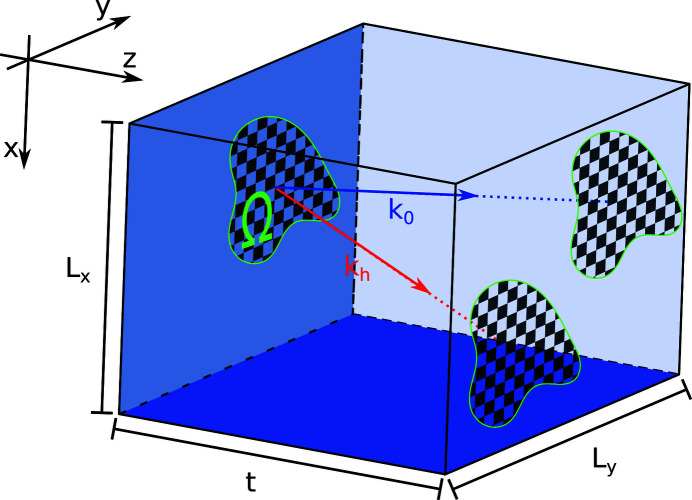
Scattering geometry inside the sample volume and the finite support of the initial condition.

These boundary conditions require that the sample grid be large enough to fit the Borrmann triangle extending from every point where the initial condition is non-zero. If the initial condition is only non-zero on a domain Ω on the surface (*i.e.* at 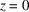 ), the direct projection of this domain along the directions of 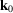 and 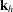 must lie within the sample grid (see Fig 1 ▸). This condition is fulfilled if the domain Ω is fully contained in the rectangle defined by

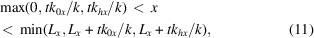




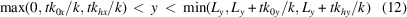

which can always be made true for a finitely bounded initial condition if the computational grid is sufficiently large.

As we are using discrete Fourier transforms we also specify a grid in ( 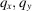 ) space, which is related to the (*x*–*y*) grid in real space. In full period frequency, this corresponds to the points

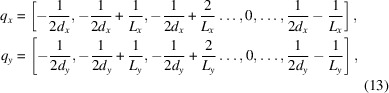

where the inclusion of the negative Nyquist frequency is specific to even numbers for 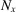 and 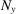 .

The complex envelope of the incident beam should be given for the entrance surface of the grid [*i.e.* 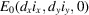 ] and the components transformed using a discrete Fourier transform (DFT) to give the initial condition in reciprocal space where the amplitude of the scattered beam is zero at the entrance surface, *i.e.* 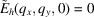 . This initial condition is constructed by appending the components of these two arrays into a single vector: 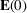 .

We can rewrite the equations (9) to the form used by exponential Runge–Kutta methods: 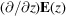 = 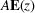 + 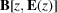 , where *A* is a diagonal matrix containing the coefficients in the square brackets of equation (9) evaluated at the reciprocal-space grid defined by equation (13) and 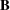 contains the convolution terms, *i.e.* the last terms of equations (9). The function is implemented by applying an inverse DFT on 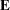 , multiplying by the scattering coefficients, that must be given on the real-space grid at the slice *z*, and transforming back to reciprocal space:

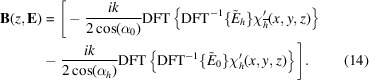




## 5. Exponential Runge methods and convergence behaviour

The Fourier-transformed TTEs can be solved by exponential Runge–Kutta methods of the type given by Hochbruck & Ostermann (2010 ▸). To test the convergence of this approach, we utilized two different exponential integrators. The first is an archetypal exponential integrator based on the explicit Euler scheme, given by

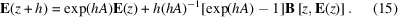

The second exponential integrator is an explicit second-order method based on Heun’s method, given by the steps (Friedli, 1978 ▸) 

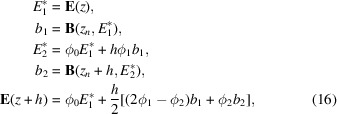

where the ϕ functions are given by 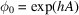 and 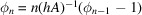 , and 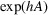 is in general the matrix-exponential which here is equal to the element-wise exponential because *A* is diagonal. We choose this scheme based on Heun’s method because it only evaluates the *B* function on the same regular intervals where the field is calculated, and therefore only requires the value of the scattering function on the same grid where the fields are evaluated.

For comparison with existing methods, we also implemented a normal finite difference method based on a recent publication by Shabalin *et al.* (2017 ▸) using the half-step finite difference for the derivatives. A derivation of this method is given in Appendix *A* [App appa].

**Figure 2:**
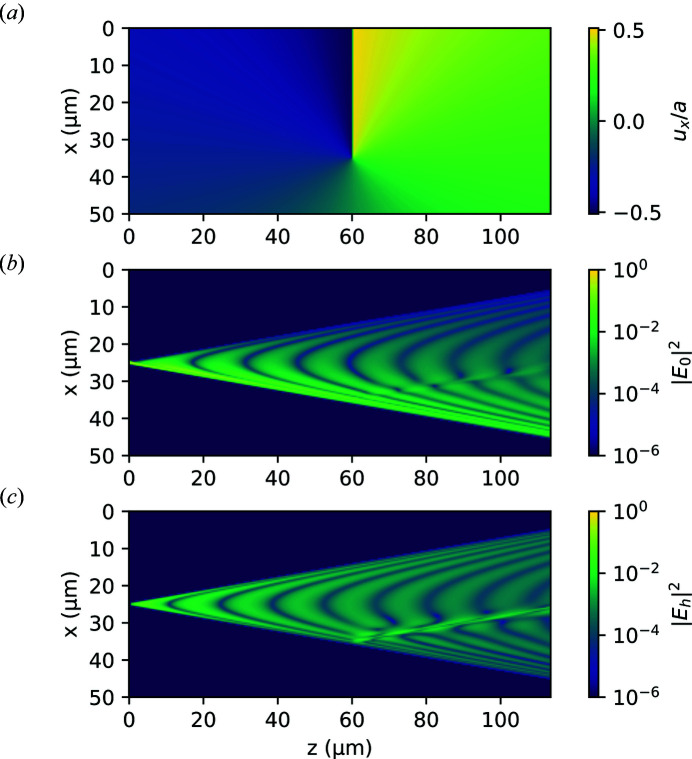
Plots of the sample and calculated fields used in the second convergence test. (*a*) Displacement field in units of the lattice constant, *a*. (*b*) Transmitted field on a logarithmic scale. (*c*) Scattered field on a logarithmic scale. The calculated fields are for the case 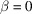 .

To evaluate the convergence behaviour of these exponential methods, we generated a virtual sample consisting of a perfect single crystal with a single edge dislocation with Burger’s vector (100) close to the path of the direct beam. Plots of the displacement field as well as the amplitudes of the converged solution are shown in Fig. 2 ▸.

The fields are simulated under low absorption and highly dynamical conditions, and we simulated only a single slice in the *y* direction with the dimensions 50 × 115 µm at a point 1 µm from the dislocation core. The incident beam is a narrow Gaussian of width σ = 0.2 µm, with parameters chosen corresponding to the (111) reflection of a diamond crystal with a molybdenum source, given as: 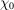 = 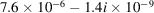 , 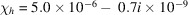 and 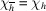 , where *i* is the imaginary unit. We set *k* = 8.85 Å^−1^ and 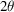 = 20°. The incident beam has a Gaussian envelope with width σ_*x*_ = 0.2 µm.

In order to accommodate the comparison with existing methods, we utilized a grid with step sizes 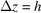 and 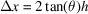 for the exponential methods and a grid with the same density of points for the normal finite difference method. Then, to check the convergence of the methods, we calculate the fields on progressively finer grids from a first grid consisting of 101 × 41 points. The error represents the deviation of the exit surface amplitudes to that computed using the normal finite difference approach on a very fine grid of 10 241 × 25 601 steps (evaluated at the points where the coarse and fine grids coincide).

**Figure 3:**
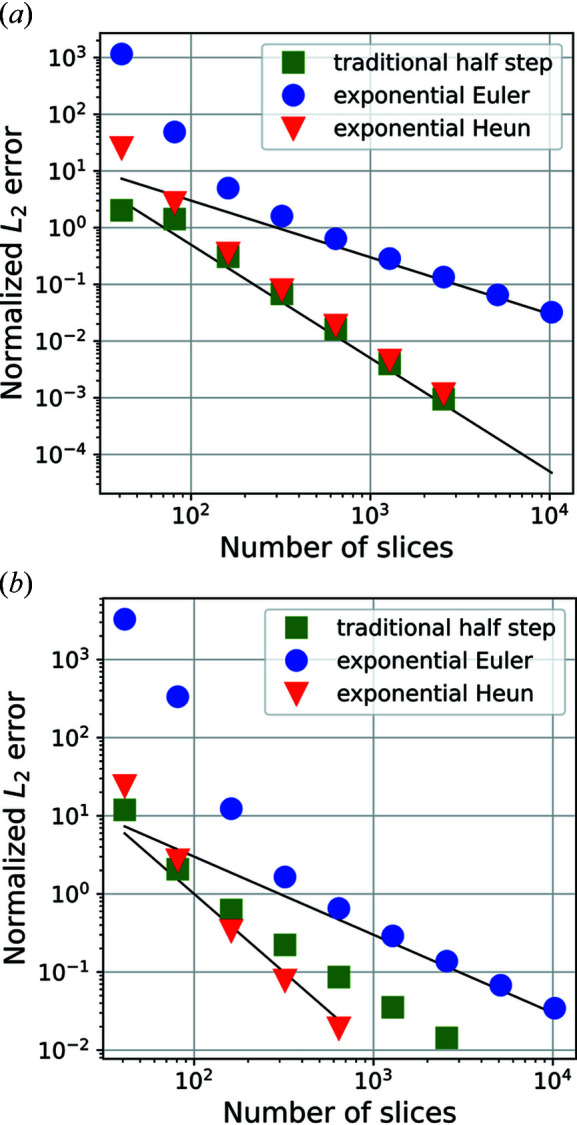
Convergence of the new exponential integrators and a traditional finite difference scheme. The black lines mark first- and second-order convergence. All errors are calculated relative to the solution using the traditional half-step method with 10 241 steps. We tested integration schemes on two different samples. One (*a*) is a perfect crystal, the other (*b*) is the edge dislocation type sample shown in Fig. 2 ▸. All calculations are in the case 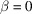 .

Fig. 3 ▸ shows the convergence of the three integration methods. While all methods show the expected convergence on a perfect sample (*a*), the traditional half-step method does not show the expected second-order convergence with the edge dislocation sample (*b*). The first-order exponential Euler method suffers from an exponential instability and only gives a qualitatively correct result when impractically small step sizes are utilized.

**Figure 4:**
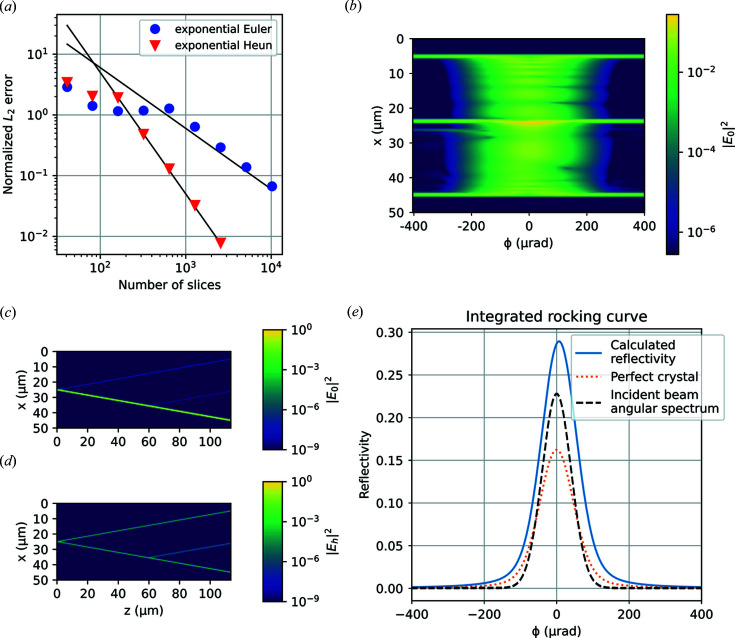
Testing of the integration algorithms for finite rocking angles. (*a*) Convergence plot for the screw-dislocation test case at 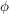 = 300 µrad. (*b*) Spatial profile of the scattered beam as a function of rocking angle. (*c*) Transmitted beam in a cross section of the crystal at 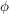 = 300 µrad. (*d*) Scattered beam in a cross section of the crystal at 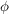 = 300 µrad. (*e*) Integrated rocking curve of the dislocation test case overlaid on the same curve calculated for a perfect crystal and the angular spectrum of the incident beam.

To demonstrate that our method is not limited only to the exact Bragg condition, we also test the convergence of our method at the tails of the rocking curve. Fig. 4 ▸(*a*) shows the convergence of the screw-dislocation test case at a rocking angle of 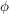 = 300 µrad, where the two methods reach the expected first- and second-order convergence. At this point, the diffraction is approximately kinematical. Figs. 4 ▸(*c*), 4 ▸(*d*) show that the transmitted beam is approximately undisturbed by the crystal and that the beam is only significantly scattered in small areas close to the surface and the dislocation. Fig. 4 ▸(*b*) shows the profile of the scattered beam at the exit surface as a function of rocking angle. We see that at low angles, the scattering is clearly dynamical and the profile shows *Pendellösung* fringes. At higher angles, the bulk of the crystal scatters less strongly and the scattering pattern is dominated by the defects. Because the divergence of the incident radiation is larger than the characteristic ‘Darwin width’ of dynamical diffraction, the crystal approximately acts as an analyser, and maps out the angular spectrum of the incident beam, which is further illustrated in Fig. 4 ▸(*e*).

## 6. Discussion and conclusion

We have described and demonstrated a finite difference scheme capable of integrating the TTEs on an orthogonal grid with few restrictions on the choice of grid. We achieve this by implicitly utilizing Fourier interpolation at the level of the individual finite difference step. The method results in approximately the same error as the traditional half-step finite difference scheme.

The method utilizes FFTs (fast Fourier transforms) at each step and has to perform in total four 2D Fourier transforms of the entire sample volume (the Heun method makes two evaluations of the 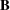 function at each step), which is expected to have a computational cost compared with the existing methods. In certain geometries, these may be replaced by 1D Fourier transforms. Our experience here, using unoptimized code, is that this increase amounts to about a factor of 4, which we believe should be unimportant in most cases of practical interest.

The ability to freely choose the computational grid makes implementation of this approach easier, especially when it needs to be combined with other numerical modelling methods, for example if the input for either the crystal microstructure or the incident field is given by a numerical simulation, or if the scattered fields should be propagated through image-forming optics.

## Supplementary Material

Python3 implementation of the described algorithm. DOI: 10.1107/S2053273322004934/iv5022sup1.exe

## Figures and Tables

**Figure 5:**
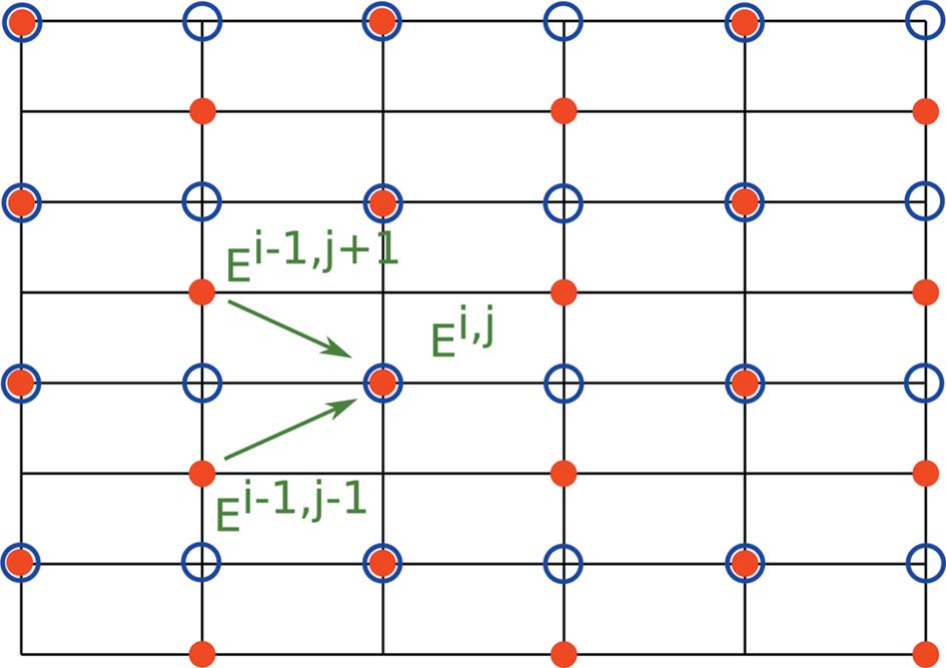
Computational grids used for the traditional half-step approach marked with red dots and for the exponential integrators marked with blue circles. The recurrence relation for the half-step method is drawn with green arrows.

## Footnotes

1 The choice of  is arbitrary, but leads to different versions of the TTEs; the other common choice is for  to be the wavevector of the refracted wave inside the crystal.

## Appendix A Traditional finite difference scheme

For comparison with the exponential integrators presented in this paper, we also present calculations performed with a second-order implicit finite difference scheme based on a central difference estimate for the derivatives, which is often applied for dynamical scattering calculations. The derivation here follows the one given by Shabalin *et al.* (2017 ▸) with small changes to the notation.

We limit our attention to a symmetric geometry defined by

Starting from equation (5) we introduce the coordinates

and arrive at a well known form of the TTE (suppressing the *y* dependence):

To avoid truncation errors due to the complex rotation caused by the  terms, we introduce the scaled fields  and  . Plugging in and simplifying some terms gives:

We now introduce a rectangular grid in the original  coordinates with step size *h* in the *z* direction and  in the *x* direction. With this choice of grid a subset consisting of every second grid point constitutes a sheared grid aligned with the  and  directions with both step sizes equal to  . This allows us to calculate the fields using the finite difference methods and the exponential integrators on grids with the same density of grid points and that coincide on every second plane. We therefore have to choose a grid with an odd number of grid points in the *x* direction so that we can compare the result on the final slice (see Fig. 5 ▸).

We denote the discretized envelope fields by  . The recurrence relation is obtained by the centred first-order approximation for the derivatives and a similar centred approximation for the right-hand sides in equation (20) to arrive at the equations

where

and

Introducing the constant  we can finally write

which are the implicit recurrence relations used in the calculations. Furthermore, we need the boundary conditions, that the fields are both zero at the top and bottom surfaces:  .

### A1. Availability

A Python3 implementation of the described algorithm is available at https://github.com/Multiscale-imaging/dynamical_diffraction and is given as supporting information.
